# Ammonium Trifluoroacetate-Mediated Synthesis of 3,4-dihydropyrimidin-2(1*H*)-ones

**DOI:** 10.5402/2011/273136

**Published:** 2011-11-13

**Authors:** Chandran Raju, R. Uma, Kalaipriya Madhaiyan, Radhakrishnan Sridhar, Seeram Ramakrishna

**Affiliations:** ^1^Pachaiyappa's College, University of Madras, Aminjikarai, Chennai 600 029, India; ^2^HEM Laboratories, National University of Singapore, Singapore; ^3^King Saud University, Riyadh 11451, Saudi Arabia

## Abstract

A simple and economic synthesis of 3,4-dihydropyrimidin-2(1*H*)-ones using ammonium trifluoroacetate as catalyst and as solid support is accomplished. Easy workup procedure for the synthesis of title compounds is well arrived at and is well documented.

## 1. Introduction

Three component coupling reactions are very efficient and simple methodology for the synthesis of dihydropyridines [1, 2] and dihydropyrimidine derivatives [3]. Biginelli compounds and their analogues have been reported to possess a wide variety of pharmaceutical and therapeutic properties [4–11]. Though the first report on Biginelli reaction came in the 19th century, the research on dihydropyrimidines is not fully saturated because of their biological application as antihypertensive agents and calcium channel blockers [9–11]. Moreover, monastrol, a dihydropyrimidine derivative, is much exploited because of its extensive application as a cell permeable small-molecule inhibitor of the mitotic kinesin, Eg5 [12].

There are many reports for the synthesis of 3,4-dihydropyrimidin-2(1*H*)-ones using Lewis acid catalysts such as InCl_3_ [13], LaCl_3_·7H_2_O [14], Yb(OTf)_3_ [12], Mn(OAc)_3_·2H_2_O [15], Cu(OTf)_2_ [16], heteropolyacids [17], and so forth [18–30]. Phenyl boronic acid [31] was reported to catalyse the Biginelli reaction in acetonitrile solvent under refluxing conditions for 18 h. Ammonium chloride [32] solid-supported solvent-free synthesis of 3,4-dihydropyrimidin-2(1*H*)-ones at 100°C is also reported. Green approach via polystyrene sulfonic acid [33] is also reported under microwave heating at 80°C and via TaBr_5_ [34] catalyst at 75°C. 

## 2. Results and Discussion

**Scheme 1:**
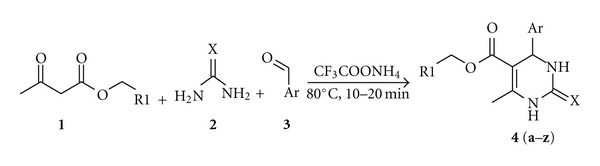


In order to overcome the strong acidic conditions, higher temperature conditions, increased reaction times, unsatisfactory yields, and complicated workup procedures, we optimized and herein we disclose a simple protocol for the synthesis of the title compounds in higher yields employing ammonium trifluoroacetate as catalyst. The role of the same as catalyst in organic synthesis is relatively less explored. The catalyst effectively imparts the acidity that catalyzes the three-component coupling at 80°C in 10 to 20 min with good to excellent yields (Scheme 1). 

Further ammonium trifluoroacetate is employed as solid support for 3,4-dihydropyrimidin-2(1*H*)-ones synthesis. The reaction mixture after completion forms the product as solid which is given water wash to get rid of the solid support. The solid was again given aqueous ethanol wash to drive off other organic impurities to obtain pure 3,4-dihydropyrimidin-2(1*H*)-ones in quantitative yield (Table 1).

 The method is worked and optimized not only for aromatic aldehydes but also for functional aromatic aldehydes (Scheme 1). Aldehydes with both the electron withdrawing and electron donating substituents are experimented under the neat reaction condition. From the results it is evident that the reaction condition or the catalyst did not affect the reactivity of electron withdrawing or electron releasing substituents in the aldehyde moiety. Further, the hetero-aromatic systems (Table 1, **4s**–**4z**) are also explored with the trifluoroacetate ammonium solid-supported protocol so as to generalize the condition for every system. Compared to the aromatic systems the heteroaromatic aldehydes are less yielding in less reactive aldehyde cases. In order to optimize the reaction condition several attempts (Table 2) were made to arrive at the successful solid-supported method. 

The versatility of ammonium trifluoroacetate is clear from the table that it affects good to excellent yield of 3,4-dihydropyrimidin-2(1*H*)-ones in both ethanol and acetonitrile at higher temperatures. The final solid-supported approach excels all the other methods giving quantitative conversion of the starting materials to 3,4-dihydropyrimidin-2(1*H*)-ones in short time. Further, the procedure avoids use of solvents for extraction, ensures safety, and lessens pollution. Decreased reaction times are also realized due to the increased reactivity of the reactants under neat condition as compared to the solvent-mediated conditions.

## 3. Conclusion

Herein we have achieved our ultimatum to obtain the Biginelli compounds through solvent free approach, in short reaction time, employing economic, weekly acidic catalyst cum solid support adopting an easy workup procedure. The synthesis and antihypertensive/calcium channel activity of novel hetero aryl substituted 3,4-dihydropyrimidin-2(1*H*)-ones through this generalized protocol will be our future aim.

## 4. Experimental Section

### 4.1. General Procedure for One-Pot Synthesis of 3,4-dihydropyrimidin-2(1*H*)-ones

A mixture of aldehyde (5 mmol), *β*-diketo ester (5 mmol), urea/thiourea (7.5 mmol) and ammonium trifluoro acetate (50 mmol) was taken in a vial and heated as neat at 80°C for 20 to 30 min. After cooling, solid formed was filtered and washed with cold water (2 × 10 mL) followed by diethyl ether, if necessary recrystallized from ethanol or ethyl acetate to afford pure product. Compound-**4b:** ^1^H NMR (300 MHz, DMSO-d_6_): *δ* 9.19 (s, 1H), 7.73 (brs, 1H), 7.23 (t, 1H), 6.81–6.76 (m, 3H), 5.10 (s, 1H), 3.99 (q, 2H, *J =* 7 Hz), 3.70 (s, 3H), 2.22 (s, 3H), 1.11 (t, 3H, *J =* 7.08 Hz). ^13^C NMR (75 MHz, DMSO-d_6_): *δ* 165.8, 159.7, 152.6, 148.9, 146.8, 130.0, 118.7, 112.8, 112.6, 99.6, 59.7, 55.4, 54.2, 18.2, 14.6. IR (KBr): 3240, 3104, 2931, 1704, 1649, 1330, 1091 cm^−1^. LC/MS: *m/z* 291 (M + H^+^). Compound-**4c:** ^1^H NMR (300 MHz, DMSO-d_6_): *δ* 13.2 (brs, 1H), 9.25 (brs, 1H), 7.85–7.79 (m, 3H), 7.46 (m, 2H), 5.19 (s, 1H), 3.97 (q, 2H, *J = *7 Hz), 2.24 (s, 3H), 1.10 (t, 3H, *J =* 7 Hz). ^13^C NMR (75 MHz, DMSO-d_6_): 167.6, 165.6, 152.4, 149.1, 145.8, 131.3, 131.2, 129.2, 128.7, 127.7, 99.4, 59.7, 54.3, 18.3, 14.5. IR (KBr): 3216, 3098, 2980, 2930, 2530, 1694, 1655, 1607, 1455, 1290, 1092, 764, 616, 515 cm^−1^. LC/MS: *m/z* 303 (M − H^+^). Compound-**4d:** ^1^H NMR (300 MHz, DMSO-d_6_): *δ* 9.36 (brs,1H), 8.13 (m, 1H), 8.06 (s, 1H), 7.89 (s, 1H), 7.70–7.61 (m, 2H), 5.29-5.28 (d, 1H, *J* = 3.18 Hz), 4.02–3.94 (m, 2H), 2.25 (s, 3H), 1.08 (t, 3H, *J* = 7 Hz). ^13^C NMR (75 MHz, DMSO-d_6_): 165.5, 152.2, 149.8, 148.2, 147.5, 133.4, 130.7, 122.8, 121.5, 98.7, 59.8, 54.0, 18.3, 14.5. IR (KBr): 3330, 3218, 3110, 2964, 1709, 1630, 1526, 1456, 1419, 1346, 1311, 1223, 1088, 1004, 813, 688 cm^−1^LC/MS: *m/z* 306 (M + H^+^). Compound-**4e:** ^1^H NMR (300 MHz, DMSO-d_6_): *δ* 9.20 (s, 1H), 7.74 (brs, 1H), 7.31–7.20 (m, 5H), 5.13 (d, 1H, *J* = 3.42 Hz), 3.51 (s, 3H), 2.23 (s, 3H). ^13^C NMR (75 MHz, DMSO-d_6_): 166.3, 152.6, 149.1, 145.1, 128.9, 127.7, 126.6, 99.5, 54.2, 51.2, 18.3. IR (KBr): 3446, 3333, 3222, 2950, 1696, 1667, 1437, 1349, 1239, 1094, 792, 698, 520, 458 cm^−1^ LC/MS:*m/z* 247 (M + H^+^). Compound-**4f:** ^1^H NMR (300 MHz, DMSO-d_6_): *δ* 10.33 (s, 1H), 9.65 (brs, 1H), 7.36–7.19 (m, 5H), 5.16 (d, 1H, *J* = 3.6 Hz), 4.03 (q, 2H), 2.28 (s, 3H), 1.10 (t, 3H). ^13^C NMR (75 MHz, DMSO-d_6_): 174.7, 165.6, 145.5, 143.9, 129.0, 128.1, 126.8, 101.2, 60.0, 54.5, 17.6, 14.5. IR (KBr): 3328, 3174, 3106, 2982, 1671, 1573, 1467, 1422, 1327, 1197, 1117, 1026, 722 cm^−1^ LC/MS: *m/z* 277 (M + H^+^). Compound-**4g:** ^1^H NMR (300 MHz, DMSO-d_6_): *δ* 9.31 (s, 1H), 7.81–7.55 (m, 5H), 5.19 (s, 1H), 3.99–3.95 (q, 2H, *J* = 7 Hz), 2.25 (s, 3H), 1.08–1.04 (t, 3H, *J* = 7 Hz). ^13^C NMR (75 MHz, DMSO-d_6_): *δ* 165.5, 158.1, 152.2, 149.8, 146.8, 131.8, 131.2, 130.5, 119.2, 111.7, 98.6, 59.8, 54.1, 18.3, 14.5. IR (KBr): 3345, 2967, 2228, 1677, 1426, 1097, 793 cm^−1^ LC/MS: *m/z* 286 (M + H^+^). Compound-**4h:** ^1^H NMR (300 MHz, DMSO-d_6_): *δ* 9.15 (s, 1H), 7.68 (s, 1H), 7.22–7.17 (m, 1H), 7.05–7.00 (m, 3H), 5.09 (brs, 1H), 4.00–3.93 (q, 2H, *J* = 7 Hz), 2.27 (s, 3H), 2.23 (s, 3H), 1.11–1.06 (t, 3H, *J* = 7 Hz). ^13^C NMR (75 MHz, DMSO-d_6_): *δ* 165.8, 152.6, 148.7, 145.3, 137.8, 128.8, 128.3, 127.3, 123.8, 99.7, 59.6, 54.4, 21.6, 18.2, 14.5. IR(KBr): 3220, 3100, 2980, 1699, 1646, 1220, 1085, 793 cm^−1^ LC/MS: *m/z* 275 (M + H^+^). Compound-**4i:** ^1^H NMR (300 MHz, DMSO-d_6_): *δ* 9.25 (s, 1H), 7.69 (s, 1H), 7.28–7.13 (m, 4H), 5.44 (brs, 1H), 3.91–3.89 (q, 2H, *J* = 7 Hz), 2.25 (s, 3H), 1.06–0.99 (t, 3H, *J* = 7 Hz). ^13^C NMR (75 MHz, DMSO-d_6_): *δ* 165.6, 161.4, 158.2, 152.0, 149.4, 132.2, 129.9, 125.0, 116.0, 97.9, 59.5, 49.1, 18.2, 14.3. IR (KBr): 3345, 3212, 3099, 2969, 1685, 1220, 1097, 749, 639 cm^−1^. LC/MS: *m/z* 277 (M − H^+^). Compound-**4j:** ^1^H NMR (300 MHz, DMSO-d_6_): *δ* 10.40 (s, 1H), 9.68 (s, 1H), 7.43–7.40 (d, 2H, *J =* 9 Hz) 7.23–7.20 (d, 2H, *J =* 9 Hz), 5.16 (s, 1H), 3.54 (s, 3H), 2.28 (s, 3H). ^13^C NMR (75 MHz, DMSO-d_6_): *δ* 174.7, 166.0, 146.1, 142.6, 132.8, 129.1, 128.7, 100.5, 53.8, 51.6, 17.7. IR (KBr): 3313, 3169, 2995, 2947, 1715, 1570, 1190, 1113, 827 cm^−1^ LC/MS: *m/z* 297 (M + H^+^). Compound-**4k:** ^1^H NMR (300 MHz, DMSO-d_6_): *δ* 9.25 (s, 1H), 7.89–7.86 (m, 4H), 7.66 (s, 1H),7.49–7.41 (m, 3H), 5.31 (s, 1H), 3.96 (q, 2H, *J =* 7 Hz), 2.27 (s, 3H), 1.06 (t, 3H, *J =* 7 Hz). ^13^C NMR (75 MHz, DMSO-d_6_): *δ* 165.8, 152.5, 149.0, 142.6, 133.1, 132.8, 128.7, 128.3, 127.9, 126.7, 126.4, 125.3, 125.0, 99.5, 59.6, 54.7, 18.3, 14.5. IR (KBr): 3223, 3102, 2932, 1705, 1648, 1428, 1321, 1284, 1228, 1086, 1020, 801, 755, 601 cm^−1^. LC/MS: *m/z* 311 (M + H^+^). Compound-**4l:** ^1^H NMR (300 MHz, DMSO-d_6_): *δ* 8.7 (brs, 1H), 7.23–7.20 (m, 4H), 7.04–7.02 (m, 2H), 4.30 (brs, 1H), 3.99 (m, 2H), 2.66 (d, 2H, *J =* 4.41 Hz), 2.04 (s, 3H), 1.17 (t, 3H, *J =* 7 Hz). ^13^C NMR (75 MHz, DMSO-d_6_): *δ* 165.8, 160.0, 152.9, 149.4, 137.5, 130.2, 128.2, 126.6, 98.5, 59.5, 52.1, 42.9, 17.9, 14.6. IR (KBr): 3441, 3334, 3248, 2982, 1701, 1646, 1460, 1312, 1230, 1098, 1026, 786 cm^−1^. LC/MS: *m/z* 275 (M + H^+^). Compound-**4m:** ^1^H NMR (400 MHz, DMSO-d_6_): *δ* 7.56 (brs, 1H), 7.21 (brd, 1H), 6.87–6.81 (m, 2H), 6.76–6.74 (dd, *J = *2.08, 6.96 Hz), 4.43–4.41 (m, 1H), 4.19 (m, 2H), 3.71 (s, 3H), 3.2 (brs, 1H), 1.7 (s, 3H), 1.22 (t, 3H, *J = *7.12 Hz). ^13^C NMR (100 MHz, DMSO-d_6_): 168.9, 154.9, 148.5, 140.3, 126.4, 120.8, 120.5, 119.2, 112.0, 83.5, 61.0, 55.8, 48.1, 24.4, 14.5. IR (KBr): 3359, 3215, 3086, 2942, 2249, 1743, 1687, 1589, 1489, 1372, 1265, 1076, 766, 597 cm^−1^. LC/MS: *m/z* 307 (M + H^+^). Compound-**4n:** ^1^H NMR (300 MHz, DMSO-d_6_): *δ* 7.69 (brs, 1H), 7.50–7.45 (m, 2H), 7.21 (bd, 1H, *J* = 3.66 Hz), 6.64 (d, 1H, *J* = 8.46 Hz), 4.49 (t, 1H, J = 4.14 Hz), 4.20–4.09 (m, 2H), 3.25 (s, 1H), 1.71 (s, 3H), 1.23 (t, 3H). ^13^C NMR (75 MHz, DMSO-d_6_): 168.6, 160.0, 154.8, 151.1, 138.1, 137.3, 128.7, 119.8, 84.0, 83.2, 78.8, 61.1, 47.6, 24.3, 14.5. IR (KBr): 3447, 3353, 3214, 3079, 2932, 1744, 1687, 1626, 1463, 1249, 1087, 1025, 909, 815, 555 cm^−1^LC/MS: *m/z* 403 (M + H^+^). Compound-**4o:** ^1^H NMR (300 MHz, DMSO-d_6_): *δ* 9.33 (s, 1H), 9.07 (s, 1H), 7.04–7.00 (m, 3H), 6.70–6.67 (d, 1H, *J* = 8.34 Hz), 5.37 (brs, 1H), 3.93–3.89 (q, 2H, *J* = 7 Hz), 2.23 (s, 1H), 1.17 (s, 9H), 1.05–1.00 (t, 3H, *J* = 7 Hz). ^13^C NMR (75 MHz, DMSO-d_6_): *δ* 166.0, 152.9, 152.7, 148.7, 140.9, 129.4, 125.3, 124.5, 115.5, 98.2, 59.3, 50.8, 33.9, 31.8, 18.1, 14.6. IR (KBr): 3382, 3283, 2958, 1678, 1629, 1219, 1003, 876, 605 cm^−1^ LC/MS: *m/z* 331 (M − H^+^). Compound-**4p:** ^1^H NMR (300 MHz, DMSO-d_6_): *δ* 11.36 (s, 1H), 9.24 (s, 1H), 8.01 (m, 1H), 7.87–7.86 (m, 1H), 7.43 (brs, 1H), 6.97–6.94 (d, 1H, *J* = 8.84 Hz), 5.45 (s, 1H), 3.92–3.89 (q, 2H, *J* = 7 Hz), 2.27 (s, 3H), 1.05–0.99 (t, 3H, *J* = 7 Hz). ^13^C NMR (75 MHz, DMSO-d_6_): *δ* 165.6, 162.1, 152.3, 149.8, 139.6, 131.5, 125.4, 124.5, 116.4, 97.1, 59.6, 50.4, 18.2, 14.4. IR (KBr): 3428, 3100, 2983, 1693, 1640, 1488, 1333, 1238, 1073, 820, 747, 639 cm^−1^ LC/MS: *m/z* 320 (M − H^+^). Compound-**4q:** ^1^H NMR (300 MHz, DMSO-d_6_): *δ* 9.41 (s, 1H), 80.5 (s, 1H), 7.91–7.84 (m, 3H), 5.37 (brs, 1H), 3.99–3.96 (q, 2H), 2.26 (s, 3H), 1.07–1.02 (t, 3H). ^13^C NMR (75 MHz, DMSO-d_6_): *δ* 165.4, 152.0, 150.3, 148.7, 131.0, 130.7, 127.5, 125.1, 98.3, 59.9, 54.0, 18.3, 14.3. IR (KBr): 3441, 3321, 1654, 1543, 1275, 1118, 896, 676 cm^−1^. LC/MS: *m/z* 395 (M − H^+^). Compound-**4r:** ^1^H NMR (300 MHz, DMSO-d_6_): *δ* 10.45 (s, 1H), 9.65 (s, 1H), 7.58–7.55 (dd, 1H, *J* = 1.5, 7.8 Hz), 7.39–7.33 (t, 1H, *J* = 7.8 Hz), 7.27–7.24 (dd, 1H, *J* = 1.5, 7.8 Hz), 5.67 (brs, 1H), 3.46 (s, 3H), 2.23 (s, 3H). ^13^C NMR (75 MHz, DMSO-d_6_): *δ* 174.44, 165.68, 146.50, 143.52, 132.47, 130.44, 129.20, 128.29, 127.0, 99.80, 52.81, 51.54, 17.59. IR (KBr): 3153, 2987, 1715, 1560, 1467, 1193, 1099, 723 cm^−1^. LC/MS: *m/z* 332 (M + H^+^). Compound-**4s:** ^1^H NMR (300 MHz, DMSO-d_6_): *δ* 9.33 (brs, 1H), 7.91 (brs, 1H), 7.35 (d, 1H, *J* = 4.98 Hz), 6.94–6.88 (m, 2H), 5.26 (s, 1H), 4.08 (q, 2H, *J* = 7.08 Hz), 2.20 (s, 3H), 1.17 (t, 3H, *J* = 7.11 Hz). ^13^C NMR (75 MHz, DMSO-d_6_): 165.5, 160.0, 152.7, 149.2, 127.1, 125.1, 123.9, 100.3, 59.8, 49.8, 18.1, 14.6. IR (KBr): 3446, 3336, 2983, 1628, 1457, 1315, 1231, 1157, 1025, 710, 556 cm^−1^ LC/MS: *m/z* 267.1 (M + H^+^). Compound-**4t:** ^1^H NMR (300 MHz, DMSO-d_6_): *δ* 9.19 (brs, 1H), 7.76 (brs, 1H), 7.46–7.43 (m, 1H), 7.13 (brs, 1H), 6.98 (d, 1H, *J* = 4.92 Hz), 5.20 (bd, 1H, *J* = 3.21 Hz), 4.04 (q, 2H, *J* = 7.1 Hz), 2.20(s, 3H), 1.16 (t, 3H, *J* = 7.05 Hz). ^13^C NMR (75 MHz, DMSO-d_6_): 165.7, 153.0, 148.9, 148.9, 146.2, 127.1, 127.0, 126.6, 121.2, 99.9, 59.7, 49.8, 18.2, 14.6. IR (KBr): 3241, 3108, 2980, 1702, 1649, 1461, 1425, 1369, 1291, 1093, 687, 513 cm^−1^ LC/MS: *m/z* 267 (M + H^+^). Compound-**4u:** ^1^H NMR (300 MHz, DMSO-d_6_): *δ* 9.25 (s, 1H), 8.59–8.57 (d, 1H, *J* = 4.5 Hz), 7.96–7.91 (t, 1H, *J* = 7.6 Hz), 7.70 (s, 1H), 7.44–7.40 (m, 2H), 5.28 (brs, 1H), 3.97–3.91 (q, 2H, *J* = 7 Hz), 2.22 (s, 3H), 1.09–1.01 (t, 3H, *J* = 7 Hz). ^13^C NMR (75 MHz, DMSO-d_6_): *δ* 165.6, 161.7, 152.3, 149.9, 147.8, 139.5, 123.9, 122.4, 97.6, 59.6, 55.6, 18.4, 14.5. IR (KBr): 3209, 3081, 2947, 1698, 1650, 1068, 814 cm^−1^LC/MS: *m/z* 262 (M + H^+^). Compound-**4v:** ^1^H NMR (300 MHz, DMSO-d_6_): *δ* 9.16 (s, 1H), 7.64–7.54 (m, 2H), 7.36 (s, 1H), 6.32 (s, 1H), 5.08 (brs, 1H), 4.06–4.02 (q, 2H, *J* = 7 Hz), 2.19 (s, 3H), 1.18–1.13 (t, 3H, *J* = 7 Hz). ^13^C NMR (75 MHz, DMSO-d_6_): *δ* 165.7, 153.2, 149.1, 144.0, 139.0, 129.5, 109.6, 99.5, 59.7, 46.3, 18.1, 14.7. IR (KBr): cm^−1^3236, 3110, 2984, 1697, 1646, 1210, 1092, 773. LC/MS: *m/z* 251 (M + H^+^). Compound-**4w:** ^1^H NMR (300 MHz, DMSO-d_6_): *δ* 9.39 (brs, 1H), 7.99 (brs, 1H), 7.72-7.71 (d, 1H, *J* = 3.21 Hz), 7.62-7.61 (d, 1H, *J* = 3.21 Hz), 5.47 (brs, 1H), 4.08–4.01 (q, 2H, *J* = 7 Hz), 2.22 (s, 3H), 1.15–1.09 (t, 3H, *J* = 7 Hz). ^13^C NMR (75 MHz, DMSO-d_6_): *δ* 173.3, 165.3, 152.5, 150.4, 142.9, 120.7, 98.5, 59.9, 52.0, 18.2, 14.6. IR (KBr): 3204, 3074, 2855, 1692, 1632, 1214, 1088, 944, 752 cm^−1^ LC/MS: *m/z* 268 (M + H^+^). Compound-**4x:** ^1^H NMR (300 MHz, DMSO-d_6_): *δ* 10.34 (s, 1H), 9.62 (brs, 1H), 9.02-9.01 (d, 1H, *J* = 2.0 Hz), 7.39 (d, 1H, *J* = 2.0 Hz), 5.34 (brs, 1H), 3.56 (s, 3H), 2.24 (s, 3H). ^13^C NMR (75 MHz, DMSO-d_6_): *δ* 175.3, 165.9, 158.0, 155.4, 146.2, 116.0, 100.0, 51.5, 50.7, 17.7. IR (KBr): 3338, 3213, 2947, 1655, 1567, 1443, 736 cm^−1^ LC/MS: *m/z* 270 (M + H^+^). Compound-**4y:** ^1^H NMR (300 MHz, DMSO-d_6_): *δ* 11.2 (brs, 1H), 8.96 (s, 1H), 6.72 (s, 2H), 4.93 (s, 1H), 4.05–3.98 (q, 2H, *J* = 7 Hz), 2.19 (s, 3H), 1.14–1.09 (t, 3H, *J* = 7 Hz). IR (KBr): 3358, 3165, 3039, 2980, 2900, 2810, 1654, 1511, 1207, 1016, 818, 657 cm^−1^ LC/MS: *m/z* 251 (M + H^+^). Compound-**4z:** ^1^H NMR (300 MHz, DMSO-d_6_): *δ* 9.13 (s, 1H), 7.62–7.59 (m, 2H), 7.37 (d, 1H, *J* = 8.04 Hz), 7.12 (t, 1H, *J* = 8.04 Hz), 7.05–7.00 (m, 2H), 5.42 (s, 1H), 3.97–3.92 (q, 2H, *J* = 7 Hz), 3.70 (s, 3H), 2.25 (s, 3H), 1.10 (t, 3H, *J* = 7 Hz). ^13^C NMR (75 MHz, DMSO-d_6_): 165.9, 160.0, 152.9, 147.9, 137.3, 127.5, 125.8, 121.5, 119.7, 117.9, 110.0, 99.7, 59.5, 47.3, 32.7, 18.2, 14.6. IR (KBr): 3443, 3349, 3251, 2935, 2815, 1696, 1640, 1465, 1375, 1218, 1086, 786, 555 cm^−1^ LC/MS: *m/z* 314 (M + H^+^).

## Figures and Tables

**Table 1:** General synthesis of ammonium trifluoroacetate-mediated dihydropyrimidines.

Compound	Ar	R1	X	Time (min)	Yield (%)^a,b^	Mp (°C)
**4a**	Phenyl	CH_3_	O	10	98	200–202
**4b**	3-Methoxy phenyl	CH_3_	O	12	95	224-225
**4c**	3-Carboxyphenyl	CH_3_	O	15	90	291–293
**4d**	3-Nitrophenyl	CH_3_	O	10	85	231–233
**4e**	Phenyl	H	O	10	92	190-191
**4f**	Phenyl	CH_3_	S	20	83	209–211
**4g**	3-Cyanophenyl	CH_3_	O	25	78	236-237
**4h**	3-Methyl phenyl	CH_3_	O	20	95	233-234
**4i**	2-Fluorophenyl	CH_3_	O	18	70	235-236
**4j**	4-Chlorophenyl	H	S	15	75	138-139
**4k**	2-Naphthyl	CH_3_	O	10	90	210–212
**4l**	Benzyl	CH_3_	O	20	85	176–178
**4m**	2-Hydroxy-5-methoxy phenyl	CH_3_	O	28	73	241-242
**4n**	2-Hydroxy-5-iodophenyl	CH_3_	O	60	55	170-171
**4o**	2-Hydroxy-5-*t*-butyl phenyl	CH_3_	O	8	70	220–222
**4p**	2-Hydroxy-5-nitrophenyl	H	S	18	82	181-182
**4q**	3,5-Bis-trifluoromethyl phenyl	CH_3_	O	35	60	209-210
**4r**	2,3-Dichlorophenyl	H	S	18	70	182–184
**4s**	2-Thienyl	CH_3_	O	12	78	206–208
**4t**	3-Thienyl	CH_3_	O	15	70	234-235
**4u**	2-Pyridyl	CH_3_	O	25	85	183–185
**4v**	3-Furyl	CH_3_	O	20	45	206-207
**4w**	2-Thiazolyl	CH_3_	O	20	60	215-216
**4x**	4-Thiazolyl	H	S	15	55	270–273
**4y**	2-Imidazolyl	CH_3_	O	30	35	258–260
**4z**	1-Methyl-indol-3-yl	CH_3_	O	40	50	199–201

^a^Isolated yield.

^b^All the target molecules were characterized with IR, LCMS, ^1^H NMR, and ^13^C NMR.

**Table 2:** Conditions attempted for the ammonium trifluoroacetate-mediated synthesis^a^.

Entry	Condition adopted	Time	Yield (%)
1	Ethanol/catalyst/RT	12 h	65
2	Ethanol/catalyst/80°C	5 h	80
3	Acetonitrile/catalyst/RT	10 h	83
4	Acetonitrile/catalyst/80°C	30 min	90
5	Neat/catalyst/RT	20 h	10
6	Neat/catalyst-SiO_2_/RT	20 h	15
7	Neat/catalyst/80°C	10 min	98

^a^Isolated yield.
